# The Immune Mechanisms of Severe Equine Asthma—Current Understanding and What Is Missing

**DOI:** 10.3390/ani12060744

**Published:** 2022-03-16

**Authors:** Joana Simões, Mariana Batista, Paula Tilley

**Affiliations:** 1 CIISA-Centre for Interdisciplinary Research in Animal Health, Faculty of Veterinary Medicine, University of Lisbon, 1300-477 Lisbon, Portugal; marianabatista@fmv.ulisboa.pt (M.B.); paulatilley@fmv.ulisboa.pt (P.T.); 2 Associate Laboratory for Animal and Veterinary Sciences (AL4Animals), Faculty of Veterinary Medicine, University of Lisbon, 1300-477 Lisbon, Portugal; 3 Equine Health and Welfare Academic Division, Faculty of Veterinary Medicine, Lusófona University, Campo Grande 376, 1749-024 Lisbon, Portugal; 4 Basic Sciences Academic Division, Faculty of Veterinary Medicin, Lusófona University, Campo Grande 376, 1749-024 Lisbon, Portugal

**Keywords:** severe equine asthma, immunology, genetic, neutrophils, horse

## Abstract

**Simple Summary:**

Severe equine asthma (sEA) is a highly prevalent respiratory disease affecting adult horses. Affected horses present with cough, nasal discharge and increased respiratory effort at rest. Although a complex diversity of genetic and immunological pathways contribute to the disease, these remain to be fully understood. Several studies have reported the role of inflammatory mediators and of some cells found in sEA airway inflammation. However, the reported results revealed some inconsistencies between studies. A better understanding of sEA’s genetics and detailed immunology is fundamental in order to characterize the underlying mechanisms involved in the disease’s occurrence and to establish an adequate therapy and a precise prognosis. This review examines some literature findings on the genetic and immunology of sEA and discusses further research areas.

**Abstract:**

Severe equine asthma is a chronic respiratory disease of adult horses, occurring when genetically susceptible individuals are exposed to environmental aeroallergens. This results in airway inflammation, mucus accumulation and bronchial constriction. Although several studies aimed at evaluating the genetic and immune pathways associated with the disease, the results reported are inconsistent. Furthermore, the complexity and heterogeneity of this disease bears great similarity to what is described for human asthma. Currently available studies identified two chromosome regions (ECA13 and ECA15) and several genes associated with the disease. The inflammatory response appears to be mediated by T helper cells (Th1, Th2, Th17) and neutrophilic inflammation significantly contributes to the persistence of airway inflammatory status. This review evaluates the reported findings pertaining to the genetical and immunological background of severe equine asthma and reflects on their implications in the pathophysiology of the disease whilst discussing further areas of research interest aiming at advancing treatment and prognosis of affected individuals.

## 1. Introduction

Severe equine asthma (sEA) is a naturally occurring chronic respiratory disease [1], affecting up to 20% of adult horses in the Northern hemisphere [2]. Disease develops upon exposure of genetically susceptible individuals to environments with high concentrations of airborne respirable particles, capable of inducing airway inflammation [3]. A vast array of antigens have been implicated in the etiology of sEA and it is thought that airway inflammation results from the synergistic effect of multiple allergens [4], to which individuals are susceptible in unique ways. This disease has been mostly associated with hay feeding and stabling, being termed as stable-associated sEA, but summer pasture-associated sEA also occurs [1,5,6]. Fungal spores, bacterial endotoxins, forage and storage mites, microbial toxins, peptidoglycans, proteases, pollen and plant debris, as well as inorganic particles trigger clinical signs of disease [5,6,7,8,9,10]. Several fungi (>50 species), especially *Aspergillus fumigatus*, have been widely recognised as significant risk factors for sEA [11,12,13]. Recent research by White and colleagues has uncovered the potential role of novel allergens including new species of fungi, mites, pollen, and arthropods, but also that of latex proteins [5], which hadn’t yet been clearly associated with the disease.

During disease exacerbation affected horses develop cough, nasal discharge and increased respiratory effort at rest [1,14,15], due to neutrophil recruitment, mucus plugging, bronchospasm and airway remodeling [16,17].

Severely asthmatic horses are usually managed through antigen avoidance and the use of corticosteroids and bronchodilators to reduce airway inflammation, bronchoconstriction and improve lung function [18]. However, some horses are unresponsive to corticosteroid treatment posing a challenge to clinicians. Thus, the identification of causal antigens and the development of antigen screening tests is fundamental and will enable a personalized treatment approach using specific immunotherapy [5,19].

Disease diagnosis is mostly based on history, clinical signs, and bronchoalveolar lavage fluid (BALF) differential cytology. Although lung function testing can accurately detect sEA, such equipment is unavailable to most field practitioners [1,14,20]. The genetic and immunological mechanisms associated with this disease are complex and heterogenous, implicating the activation of different inflammatory pathways [21,22,23]. Currently there is a need to better characterize the immune events leading to the occurrence and persistence of airway inflammation as this will help clinicians in determining the best treatment approach and in providing an accurate prognosis. Moreover, the development of novel ancillary diagnostic tests and therapeutic targets are required for early diagnosis of sEA and total resolution of airway inflammation in refractory cases.

Because sEA shares many similarities to its human counterpart, the horse is considered a good model for the study of the non-allergic and late on-set asthma phenotypes, since disease occurs naturally and sample collection can be easily performed [24]. Thus, further contributions to the disease’s characterization will benefit both horses and humans alike.

In the present systematic review, the intention was to combine the data published over the last twenty years on the immune mechanisms which have been identified, described, and associated with sEA, in order to help researchers and clinicians to better understand this highly prevalent respiratory disease. We must recognize the limitations of this systematic review, as it does not reanalyze available data as a metanalysis would. Nevertheless, we believe that, by assembling the existing information, we can contribute to the identification of knowledge gaps to address in future scientific discussions and research projects, hopefully leading to further enlightening of the immune mechanisms of sEA.

## 2. Genetic Background

Although sEA’s heritability has been shown in several horse breeds, and a familial aggregation has long been ascertain, external factors, such as environment, increase the likelihood of expressing the disease [3,21,22].

The chromosome region ECA13 has been associated with sEA in one family of Swiss Warmbloods, while region ECA15 has been implicated in a different family of the same breed. The inheritance mode differed between both families, being autosomal recessive in the first family and autosomal dominant in the second [25]. Additionally, in the first family of horses the Interleukin 4 receptor (IL-4R) gene and its neighboring regions in ECA13 appeared to contribute to disease in some individuals [26,27,28]. In humans, polymorphic differences in the Interleukin 4 receptor α chain (IL4Rα) gene play an important role in the development of asthma, since they induce the isotopic switch to immunoglobulin E (IgE) and the differentiation of T-helper type 2 (Th2) lymphocytes [29,30].

Racine and colleagues described an interaction between IL-4R and products of the *SOCS5* gene, which may influence the molecular cascades involving nuclear factor (NF)-κB [31]. The gene coding for SOCS5 is located in the ECA15 chromosome region, and it is predominantly expressed by Th1 cells while further inhibiting Th2 differentiation. The inhibitory effect of SOCS5 on IL-4 signaling contributes to the non-Th2 cytokine profile observed in human non-allergic asthma [32], and may explain further similarities between both species.

In a genome wide association study (GWAS), the gene responsible for the TXNDC11 protein, also located in the ECA13 region, has been linked to sEA [33]. In humans, TXNDC11 controls the production of hydrogen peroxide in the respiratory epithelium [34], as well as the expression of MUC5AC mucin, which has been shown to play a significant role on airway hyperreactivity in mice [35]. In sEA-affected horses MUC5AC is upregulated, thus contributing to the mucus plugging observed in the disease [36]. 

The analysis of genomic copy number variants did not reveal any relevant variant regions which could be associated with the sEA, although a copy number loss was reported on chromosome 5 involving the gene NME7 [37]. The expression of this gene is necessary for ciliary function in the lungs and may be involved in sEA, since in knockout mice it induces primary ciliary dyskinesia [38]. Also, using RNA sequencing technique, a single point substitution was detected in the PACRG and RTTN genes in asthmatic horses, predictively altering their proteins, which are related to ciliary function [39]. 

In a gene set enrichment analysis of the bronchial epithelium after hay dust exposure, asthmatic horses presented upregulated genes of the E2F transcription factor family, which contribute to cell cycle regulation. Thus, asthmatic horses may suffer from impaired bronchial epithelial regeneration associated to subepithelial remodeling [40].

These recent studies have shown that the respiratory epithelium contributes to the immunological response observed in severely asthmatic horses.

Furthermore, an analysis of expression quantitative trait loci (eQTLs) allied with GWAS did not find a significant association between observed genetic variants and sEA, except for a disease-genetic variant in CLEC16A gene, which regulates gene expression of dexamethasone-induced protein (DEXI) [41]. This is of special importance in comparative pathology, as DEXI has also been reported in human asthma [42], although in horses it appears to not be a reliable indicator of sEA [41].

The identification and differential expression analysis of microRNAs (miRNAs) present in the serum of sEA-affected horses, showed a downregulation of miR-128 and miR-744. These findings suggest that a Th2/Th17 immunological response may characterize sEA [10,43].

Additionally, a recent work on Polish Konik horses aimed to detect the effects of inbreeding on sEA, however no effects were observed at the individual level [44]. 

Although most of these findings relate to certain families of Swiss Warmblood horses, they illustrate the complex genetic heterogeneity of sEA, which most likely results from the interaction of different genes. However, the use of such specific horse families and the likelihood of high variety of genetic background mechanisms contributing to the disease limits the application of these findings to the general equine population.

## 3. Immunological Phenotypes and Endotypes

Phenotype is the term used to describe the observable clinical characteristic of a disease, whereas endotype, a subclass of phenotype, refers to its molecular and genetic mechanism or treatment response [45]. 

As stated in the 2016 consensus, equine asthma (EA) is currently defined by two major phenotypes, which differ according to disease onset, clinical presentation and its severity—mild/moderate EA (mEA) and the already described sEA, which is the focus of this review [1]. However, phenotypes are insufficient when deciding upon the appropriate therapeutic management or determining the prognosis, which mainly depend on the immunological mechanisms of the disease.

Human asthma is usually considered to be a type 1 hypersensitivity, due to increased levels of IgE associated with a Th2 response, resulting in the recruitment of eosinophils into the airways [46]. However, an endotype which does not appear to be associated to a Th2 response has also been identified. As such, human asthma is divided into two major endotypes according to cytokine profile: Th2 and non-Th2 type asthma [47]. The Th2 type asthma is considered an allergic phenotype with the aforementioned eosinophil involvement and because its cytokine profile has been thoroughly described, several biomarkers are available for characterizing the disease and will be addressed bellow.

However, sEA is typically characterized by a neutrophilic response [1,48], and appears to not have the typical presentation of a type 1 hypersensitivity [49]. Although a Th2 cytokine profile has been described in sEA-affected horses, these animals do not display an early phase response [50,51]. However, a late phase response leading to neutrophilic bronchiolitis, associated with an increase in CD4+ T cells in the bronchoalveolar lavage fluid (BALF), has been described [50,52,53]. These features have led to the hypothesis that a type 3 hypersensitivity response, resulting in antibody-antigen complexes and activation of complement cascade, were involved in the disease’s immunology [54]. However, because sEA does not possess most of the features described in type 3 hypersensitivities, it is unlikely that this type of response accounts for the main immunological features of the disease [55].

Still the precise cytokine profile of sEA remains unclear, with a multitude of reports pointing to either a Th1, a Th2, a Th17 or a mixed mediated response. Table 1 illustrates the cytokines reported in sEA-affected horses.

Using immunohistochemistry and in situ hybridization, the expression of IL-4 and IL-5 was observed in the BALF lymphocytes of sEA-affected horses [49,53]. However, the Th2 cytokine profile of these animals was accompanied by airway neutrophilia, but not eosinophilia.

Other authors have reported an increased expression of IL-1β, IL-8, gamma-interferon (IFN-γ), tumor necrosis factor (TNF)-α, and IL-17, mainly suggesting a Th1 and/or Th17 mixed mediated response [59,60,61].

Gene expression analysis of BALF cells and bronchial epithelium of severely asthmatic horses, using reverse transcription polymerase chain reaction (RT-PCR), revealed that the expression of IL-1β, IL-8, NF-κB and toll-like receptor (TLR)4 was upregulated in these animals. Furthermore, authors reported that these findings correlated with the neutrophil percentage detected in the BALF [59].

Ainsworth and colleagues reported that during remission severely asthmatic horses exhibited an increased expression of IL-13 and despite BALF neutrophilia no differences in cytokine expression were observed 24 h after environmental challenge. However, after 5 weeks of chronic exposure to aeroallergens asthmatic horses presented increased IFN-γ and IL-8 gene expression [60].

After antigen challenge, the BALF cells of sEA-affected horses showed elevated gene expression of IL-17, IL-8 and TLR4. Gene expression of IL-8 was also increased in the bronchial epithelium, and using immunohistochemistry was tracked to the ciliated epithelium of affected horses. Additionally, stimulated peripheral blood neutrophils of asthmatic horses incubated with lipopolysaccharide (LPS) and formyl-methionyl-leucine phenylalanine (fMLP), two potent pro-inflammatory agents associated with sEA, revealed upregulated gene expression of IL-17 and TLR4 [61].

The presence of a mixed Th1/Th2 cytokine profile, involving mediators such as IL-4 and IFN-γ, has also been reported [57,58]. Disease exacerbation, post-antigen challenge, was also accompanied by elevated expression of IL-1β, TNF-α, IL-8 and IFN-γ, and treatment with fluticasone decreased mRNA expression of TNF-α [57]. Similarly, horses diagnosed with summer pasture-associated sEA developed disease exacerbation during the summer months, with increased expression of IL-13 and IFN-γ by BALF lymphocytes and CD4+ lymphocytes from peripheral blood. Furthermore, during disease remission, in the winter, these animals exhibited increased IL-4 mRNA expression [58].

The possibility of a mixed Th2/Th17 response has also been postulated [43], associated with a dysregulated Th17 cell differentiation pathway [62]. Eleven differentially expressed miRNAs (DEmiRs) were reported in the serum of asthmatic horses, compared to healthy individuals. Also, a shift towards the maturation of Th2 cells was proposed, supported by decreased levels of miR-128, which in association with decreased miR-197 and increased levels of miR-744 affects the maturation of Th cells towards a Th17 profile [43].

The analysis of the miRNAs and mRNA found in the lung tissue of sEA-affected horses supports the hypothesis of a Th17 mediated response, but also of a Th2 immune response [62]. Additionally, the upregulated miRNAs miR-142-3p and miR-223 found in asthmatic horses are also associated with severe neutrophilic asthma in humans, and with increased expression of IL-1β, IL-6 and IL-8 [63] cytokines, some of which have been associated with sEA [57,59].

Contrarily, Kleiber and colleagues reported neither a specific Th1 nor a Th2 specific response, but a downregulation of expressed cytokines (IL-4, IL-5, IL-13 and IFN-γ) in the CD4 and CD8 populations of the peripheral blood and BALF of sEA-affected horses [56], which could implicate the involvement of other pathways in the disease.

Thus, the reported results may reflect the heterogeneity of the cytokine profile involved in sEA and may imply the existence of different disease endotypes. However the interpretation of these results must necessarily take into consideration the described methodologies of the above-mentioned studies. For example, cytokine expression was investigated using distinct samples, namely BALF, bronchial and lung tissue, as well as peripheral blood. As such, results may not only reflect the inflammatory response of the examined cells, but also differences between local and systemic inflammatory responses.

With the development of transcriptomics, novel techniques for assessing the existence and relative prevalence of several RNA species have been introduced to the scientific community. This is portrayed in the reported methodologies of the aforementioned studies, where recent experiments sequence mRNA and miRNA, contrasting with the less comprehensive/detailed methods, such as traditional targeted immunohistochemistry, in situ hybridization and RT-PCR.

Additionally, the experimental design of most studies involved the exacerbation of the disease by exposing the asthmatic horse to an intense pro-inflammatory environment, using hay dust and/or by stabling the affected horses. It cannot be excluded that the experimental induction of airway inflammation may interfere to some extent with the expressed cytokine profile, especially considering individual susceptibilities to specific allergens. Therefore, this factor also needs to be taken into account when interpreting reported results.

As in human asthma, it is highly likely that sEA possesses multiple endotypes, and considering the neutrophil recruitment observed in affected horses, a Th17 mediated response is probably part of the inflammatory pathways involved in this disease. Nevertheless, more encompassing studies involving genomics, transcriptomics and proteomics are required to better define the cytokine profile of sEA and to determine therapeutic targets in affected horses, and although further confirmation is required, the reported DEmiRs may constitute novel therapeutic targets for sEA.

Severely asthmatic horses may also present an altered response to allergens, since ex vivo and in vivo hay dust stimulation revealed upregulation of several genes participating in the inflammation [10,64]. Pacholewska and colleagues reported an increased expression of CXCL13 chemokine [10] which may indicate a Th17 mediated response [65], but no evidence of a Th1 nor a Th2 response was found. Additionally, in a murine model of allergic airway inflammation increased expression of CXCL13 has been reported and its neutralization reduced allergic inflammation by decreasing lymphocytes, eosinophils, as well as the recruitment of CXCR5-bearing cells [66]. Accordingly, in humans, IL-17 expression has been associated with severe neutrophilic asthma and in horses this cytokine is responsible for the activation and persistence of neutrophils in the airways. Also, IL-17 was shown to be associated with reduced response to corticosteroids, with post-treatment persistence of IL-8 [67,68]. 

The described heterogeneity also occurs in human asthma, where one could consider the existence of three distinct phenotypes: allergic asthma, non-allergic asthma and late-onset asthma [22]. These phenotypes may also be applicable when describing sEA. In this sense the allergic asthma phenotype would be characterized by a Th2 mediated response and usually associated with other allergic diseases. In general, horses affected with sEA may also suffer from other allergic diseases such as insect bite hypersensitivity or atopy [69,70]. Interestingly, Lo Feudo and colleagues have reported the presence of a type 1 hypersensitivity in sEA-affected horses in response to intradermal allergen test, which may further support the hypothesis of an allergic phenotype [71]. Additionally, the use of skin prick tests has previously been used to identify allergic sensitization in severely asthmatic horses [19]. Similarly, evidence of a type 1 hypersensitivity to different allergens has also been described in severely asthmatic horses using allergen inhalation [72].

The non-allergic phenotype in humans is usually associated with the presence of neutrophils in the airways, a hallmark of sEA. This phenotype also reflects the involvement of a Th1 and of a Th17 response [62], which has also been described as contributing to the immune response in asthmatic horses. 

Finally, the late-onset asthma is age-associated and also occurs in sEA where affected individuals are mature adults [2,73]. This age association is thought to be the consequence of immunosenescence and inflammaging, which describe the immune and inflammatory modifications observed in geriatric patients [74,75,76], a subject extensively revised by Bullone et al. [75]. Immunosenescence is essentially a disfunction of the immune system. In horses it is usually characterized by a dysregulation of adaptative immunity associated with a lower proliferative response of T lymphocytes [77], and a decrease in mean percentage of regulatory T cells [78].

On the other hand, the term inflammaging defines the chronic inflammatory state observed in older individuals accompanied by an increased expression of inflammatory cytokines [74]. A correlation between age and IL-6 has also been described in healthy geriatric horses [79]. Also, compared to young adults, geriatric horses with colitis had higher levels of IL-6 and TNF-α [80]. It has also been described that older horses exhibit increased expression of IL-1β, IL-15, IL-18, IFN-γ and TNF-α mRNA [77,81,82]. These studies confirm that inflammaging and immunosenescence occur in geriatric horses both systemically and locally [74], and are most likely involved in the immunology of sEA, although further research is needed to clarify the age-associated changes and how they affect airway inflammatory response.

The reported differences in the immunological pathways contributing to sEA illustrate the complexity of this disease and suggest the existence of several endotypes, which converge into the same clinical phenotype. One must also consider that the methodological differences of the above mentioned studies, such as time of sample collection, natural vs. stimulated inflammatory response and duration of the disease, may have contributed to the reported variations. It is therefore fundamental that holistic studies, encompassing more exhaustive and complementary approaches, and preferably large multi-center studies can be performed to unravel sEA’s different immunological responses.

## 4. The Epithelium

The bronchial epithelium is a complex tissue composed of a single layer of ciliated columnar or cuboidal cells that intercalate with secretory cells, namely club and goblet cells [83]. These epithelial cells act as a protective barrier against foreign particles and microbes, while the mucus secreted by both epithelial secretory cells and submucosal glands, comprising a mixture of ions, proteins, lipids and large amounts of mucin glycoproteins, namely MUC5AC [36], actively contribute to this protection [84]. The relative amount of the cells that constitute this tissue is also dynamic. One example of this plasticity is the fact that the relative number of secretory cells is known to be increased in asthmatic horses in exacerbation phases when compared to controls [85]. Also, the composition of the produced mucus is disrupted in inflammatory conditions, such as sEA, and it is known that asthmatic horses, whether or not in exacerbation, have significantly decreased Salivary Scavenger and Agglutinin (SALSA) production, thus impairing innate antibacterial abilities [86].

Also present are a number of immunologically active cells, such as neutrophils, macrophages and lymphocytes. The relative number of these immune cells also varies according to inflammation status and it is known that mononuclear leukocytes [87], and mast cells [88] are increased in asthmatic patients. Airway epithelial cells are supported by a loose connective tissue [83], which is also involved in immune response and in reactive airway remodeling. In fact, recent studies have determined that the interactions between fibroblasts and the epithelium can influence airway remodeling [89].

A wide array of studies have found that the protective role of the respiratory epithelium is not exclusively mechanic, as these cells are capable of responding to offensive stimuli by secreting immunomodulatory molecules, such as chemokines, cytokines, and host defense molecules, including acute phase proteins and complement proteins [90], that regulate respiratory innate immune response. As such, studies have found that healthy bronchiolar epithelium transcribes genes of all *TLR* [90], and that *TLR3* is not only the most transcribed *TLR* in equine bronchial epithelial cells [91], but is also particularly active in response to stimulation [90,91].

Unsurprisingly, several studies have found that the epithelium of asthmatic horses responds differently to offensive stimuli than that of healthy horses. In a transcriptome analysis of endoscopically obtained biopsies, Tessier et al. found many differentially expressed genes, among which were the neutrophil chemotaxis (GO:0030593) gene set which was overrepresented in asthmatic derived samples and can explain the observed marked airway neutrophilia [64]. This study also identified the increase in *MMP-1*, *MMP-9*, *TLR4* and *IL-8* transcription as a central player in the inflammation process observed in asthmatic horses. Another relevant epithelial produced chemokine, likely involved in asthma pathophysiology and uncovered by in vitro hay dust exposure assays, is *CXCL2* [92]. CXCL2 is related to IL-8 and shares its ability to potentiate early airway neutrophilia. 

Interestingly, the differences found between the airway epithelium of asthmatic and healthy horses can account for more than the pathophysiological development of the condition, impacting also its treatment. RNA-seq analysis found a decreased expression of the rhythmic process (GO:0048511) gene set in asthmatic horses, namely *CIART*, which could disrupt the natural glucocorticoid response and promote treatment resistance [64].

As the first barrier against foreign respirable particles and microbes, the airway epithelium plays an essential role in the defense of the lung and is an integral part of the regulatory mechanisms that drive lung inflammatory processes. The continuation of the study of this tissue’s immunomodulatory properties and ways in which they can be impaired in sEA will undoubtably contribute to advance our knowledge of this pathology and find better and more efficient treatment approaches.

## 5. Alveolar Macrophages 

Alveolar macrophages (AMs) are the most common immune cells found in the lungs of healthy horses. By releasing cytokines and chemokines, such as IL-8, CXCL2 (also known as macrophage inflammatory protein-2), and TNF-α, AMs act as first respondents in the host’s defense [57,93,94,95,96]. Therefore, it is likely that these cells contribute to the pathomechanisms of sEA. 

Macrophages present different characteristics depending on the tissue where they are located [97]. Compared to peritoneal macrophages (PMs), AMs appear to possess increased responsiveness and phagocytic capacity [98]. When exposed to LPS, an important antigen implicated in sEA, AMs presented upregulation of the MyD88 and TRIF pathways [98], further highlighting the importance of these cells in the innate immune response. In comparison, only the MyD88 pathway was upregulated in PMs [98], further illustrating the differentiated role of AMs.

Depending on local microenvironment, macrophages can modify their phenotype [99,100]. The pro-inflammatory phenotype (M1) is induced by pathogen-associated molecular patterns (PAMPS) and IFN-γ [100], while the anti-inflammatory (M2) phenotype is related to wound healing and tissue repair, thus playing an important role in controlling neutrophilic inflammation through efferocytosis [101,102]. The cytokines IL-4 and IL-13 have been reported as modulators of the latter phenotype [103,104]. 

However, sEA-affected horses may lack this dynamic modulation. A recent study found that asthmatic horses at pasture had an increased expression of IL-10 in comparison to healthy controls. The authors also reported a simultaneous increased expression of IL-10 (M1 phenotype) and CD206 (M2 phenotype), suggesting the presence of a non-canonical phenotype. Furthermore, AMs maintained their responsiveness to LPS and expressed an increase in IL-8, even in the presence of IL-10, known as an inhibitor of LPS response. As such, an impaired response to IL-10 may contribute to sEA’s pathogenesis [105]. Additionally, the AMs of sEA-affected horses exposed to moldy hay presented not only increased expression of CD206 markers, but also of CD163 markers [106], further reenforcing the anti-inflammatory profile of these cells.

AMs may also contribute to the dysregulation of apoptosis described in sEA [105,107]. Apoptosis is a physiological mechanism for programed cell death and thus fundamental for inflammatory control. In asthmatic horses a delay in the apoptosis of BALF neutrophils has been reported [107,108,109]. Furthermore, Niedzwiedz and colleagues also described an increase in the early apoptotic rate of the BALF AMs [109]. 

Current knowledge on the role of macrophages is quite limited, and advances in genomic and transcriptomic analysis may help to enlighten how these cells contribute to the disease. In particular we need to understand if these cells are in fact the main agent responsible for the neutrophil recruitment and understand how they influence the inflammatory pathways associated with equine asthma. One must also consider that the M1/M2 phenotype nomenclature of macrophages is a rather simplistic concept, since it categorizes the activity of these cells into two extreme opposites—pro and anti-inflammatory. This concept is mostly based on in vitro studies (i.e., stimulation with LPS) and fails to take into consideration the local microenvironment found in vivo. Furthermore, studies have shown that macrophages can simultaneously exhibit characteristics of both phenotypes [97,110], urging the creation of a new nomenclature based on their ontogeny which more clearly encompasses the recent findings on this subject. 

AMs derive from embryonic precursors and blood monocytes, which migrate to the lung and differentiate into AMs. A recent work by Evren and colleagues describes the pathways involved in this differentiation and further defines populations of AMs based on surface cell proteins, using a humanized mouse model [111]. However, compared to humans and mice, knowledge about the precise origin of equine AMs is still vague and several conclusions are extrapolated from in vitro and in vivo studies conducted in other species.

By expressing a non-canonical phenotype associated with an altered apoptotic rate, AMs may contribute to the persistence of airway inflammation. Nonetheless, a better characterization of the equine lung macrophage population is fundamental to understand how these cells influence the inflammatory response in EA.

## 6. The Role of Neutrophils

Neutrophil recruitment, and consequent airway infiltration, is a hallmark of sEA [48,112] and has been extensively discussed in the literature [113,114]. Neutrophils contribute to the innate immune response through the phagocytosis of pathogens and the production of cytokines, chemokines and proteases, as well as reactive oxygen species (ROS) and neutrophil extracellular traps (NETs) [115,116]. Their apoptosis serves as a mechanism of controlling the action of these cells and limiting secondary tissue damage [117].

The airway neutrophilia observed in sEA and in human severe neutrophilic asthma is typically not associated with a septic inflammation. The exposure to aeroallergens and irritants results in the activation of pattern recognition receptors, namely TLR2, TLR4 and NOD2 [118,119], which interact with adaptor protein MyD88 and result in a consequent increase of cytokines and chemokines, such as IL-17, IL-8, CXCL2 and CXCL10, promoting the migration of neutrophils into the airways [120,121,122,123]. Although several studies have reported an increased expression of IL-8 in asthmatic horses during disease exacerbation [57,60,124,125], the IL-17 cytokine, whose role is further upstream in the cytokine cascade, appears to play a more significant role in neutrophil recruitment contributing to the chronicity of sEA [61,67,125,126]. IL-17 stimulates the production of CXCL1, CXCL2 [127,128,129], IL-8 and granulocyte macrophage-colony stimulating factor (GM-CSF) [127,130] and decreases neutrophil apoptosis [68]. 

Additionally, the increased expression of *TLR4* mRNA in asthmatic horses after antigen challenge [61] correlates with *IL-8* mRNA expression [131] and may further contribute to neutrophil inflammation.

NETosis is one of the mechanisms employed by neutrophils to impair infectious agents [132]. NETs are composed by nuclear DNA associated with nuclear and granule proteins and enzymes [133,134,135]. However, they are also cytotoxic and can themselves promote lung injury [135,136,137]. The IL-8 chemokine, which has been shown to be upregulated in sEA-affected horses, induces NETosis in severe human asthma [138]. Furthermore, NETs were increased in the BALF of asthmatic horses during exacerbation [139,140] and low density neutrophils (LDNs), a subpopulation of neutrophils with a greater capacity for producing NETs, have been found to be increased in the peripheral blood of humans and horses with severe asthma [141,142]. 

ROS are formed by inflammatory cells, such as neutrophils, involving NADPH oxidase [143] and contribute to cell injury and airway remodeling [144,145,146]. They are also responsible for the activation of transcription factors [147,148] and the expression of inflammatory cytokines [149]. In order to prevent oxidative injury, cells produce antioxidants. However, horses with sEA show signs of oxidant/antioxidant imbalance [150,151], including a reduction in ascorbic acid and an increase in elastase concentrations in the BALF [152,153]. Oxidative stress may also contribute to corticosteroid insensitivity in asthmatic horses. These animals maintain neutrophilic inflammation even after treatment with corticosteroids, which may be caused by the expression of the chemoattractant IL-8. In vitro it was demonstrated that oxidative stress increases the mRNA expression of *IL-8* and *IL-1β* by peripheral blood neutrophils of both healthy and asthmatic horses and that in spite treatment with dexamethasone, the upregulation of *IL-8* persisted, whilst *IL-1β* became downregulated [154]. In vivo research about the precise role of IL-8, and IL-17 will help determine if the IL-8 pathway is a suitable target for immunotherapy in asthmatic horses and humans with corticosteroid insensitivity. Additionally, sEA-affected horses may benefit from the correction of oxidative stress, although research using a more encompassing model, illustrating the microenvironment of the lungs in vivo, should be considered to evaluate the impact of oxidative stress in the inflammatory pathway.

Moreover, the bronchial epithelium is susceptible to the cytotoxic effects of neutrophil byproducts, such as ROS, exosomes and proteases [155,156], and in humans NETs are also able to induce the expression of pro-inflammatory cytokines by the epithelium [157]. In asthmatic horses the production of secretoglobulin 1A1 (SCGB1A1), a protein produced by club cells with anti-inflammatory functions, is compromised. This could be due to the decrease in the number of club cells or to a depletion of SCGB1A1 in response to chronic inflammation [139,158].

As previously mentioned, dysregulation of neutrophil apoptosis may also contribute to sEA [107,109]. This can occur through several mechanisms, such as (1) the expression of a non-canonical phenotype by AMs, which may compromise their response to efferocytes [105,159], and (2) the presence of IL-17 which increases neutrophil viability [68], thus perpetuating neutrophilic inflammation.

Neutrophils play a significant role in sEA and thus limiting their activation and increasing their clearance can improve disease resolution and limit potential complications associated with tissue injury.

## 7. Inflammatory Biomarkers 

Several biological molecules have been implicated in the inflammatory response of sEA and their identification and the knowledge of their interactions could ultimately contribute to a personalized diagnosis and disease monitoring. However, in order for a biomarker to have clinical applicability it must meet several requirements, which are summed up by the “SAVED” model. “SAVED” stands for “Superior”, “Actionable”, “Valuable”, “Economical”, and “clinically Deployable”, indicating that the new biological marker must improve current practice and patient management, as well as patient outcome, but also be cost-effective while using technology available in clinical laboratories [160]. This criteria is also being used in the development of novel biomarkers for human asthma [161].

Research on the biomarkers for equine asthma focuses mostly on two major sampling methods–BALF and peripheral blood. BALF has the advantage of better portraying the degree of airway inflammation, thus it is considered to be more representative of the disease [162]. However, BALF collection is an invasive procedure unfit to be used routinely to obtain repeated measurements or in horses with severe respiratory distress. Serum biomarkers require sampling of peripheral blood which is a far less invasive process and is usually well tolerated by horses. Unlike BALF markers, peripheral blood markers indicate systemic inflammation, rendering their application less disease-specific and making the interpretation of the obtained results more challenging [163]. Comparatively human medicine has other non-invasive alternatives, such as sputum induction [164], which is an unfeasible option in horses, and exhaled breath condensate (EBC). The applicability of exhaled breath condensate (EBC) is currently being investigated in equine asthma, although it requires specific equipment [165].

As previously mentioned, Th2 type human asthma has been thoroughly described and several biomarkers are available, thus better aiding the definition of a suitable therapeutic approach [161,166]. As such, serum IgE, fractional exhaled nitric oxide (FeNO) and blood eosinophilia are used in a clinical context to characterize disease and predict response to corticosteroids [166]. Current research is focusing on novel biomarkers which have shown promise for clinical application and require minimally invasive procedures [161], such as sputum mRNA analysis [167], serum periostin [168], exhaled breath volatile organic compounds [169], dipeptidyl peptidase-4 [170] and urinary leukotriene E4 [171]. Contrastingly, considerably less biomarkers are described for the non-Th2 type human asthma, where cytokine profile studies based on genetic, transcriptomic and proteomic analysis are considered fundamental [166,167]. Ultimately, these studies will allow optimization of personalized therapeutic targets and ensure a good clinical outcome.

Currently, only one biomarker is widely used for the diagnosis of sEA, requiring the sampling of BALF (Table 2). Since BALF neutrophilia is a hallmark of severely asthmatic horses, cutoff values have been established and are commonly used in everyday practice [1,14,172]. Unlike human Th2 type asthma, where eosinophilia occurs both in the BALF and in the peripheral blood [173], in sEA blood neutrophilia isn’t observed [1] and therefore cannot be used as a diagnostic tool. As such, current research focuses on alternative biomarkers which can substitute BALF collection, as well as consensual cutoff values, which would prove useful in identifying severely asthmatic horses in remission, monitor treatment response and contribute to precision medicine.

Acute phase proteins (APP), such as haptoglobin or serum amyloid A (SAA), are being investigated as potential indicators of disease [174,175,176]. The expression of haptoglobin was decreased in horses with summer pasture-associated sEA, compared to healthy controls [175]. This protein has been associated with airway remodeling [177], and in asthmatic children serum haptoglobin is reported to have decreased immediately after antigen challenge, although its levels did increase 24 h after exposure [178]. A different study reported that mean serum haptoglobin values were increased in severely asthmatic horses. However, authors reported that after antigen exposure an increase was also observed in the control group, suggesting that airway inflammation is reflected systemically [176].

SAA has been associated with neutrophil recruitment [179] and is increased in the serum and sputum of asthmatic people [180]. Similarly, seven days after antigen challenge sEA-affected horses presented a higher concentration of SAA [176].

**Table 2:** Biomarkers described for sEA.

Sampling Method	Biomarker	Reported Results	References
BALF			
	Neutrophils (>25% as cutoff for sEA)	Marked neutrophilia	[1]
	Haptoglobin	Decreased	[175]
	IFN-γ	Increased	[172]
	MMP-8	Increased	[181]
	MMP-9	Increased	[181,182]
Peripheral blood			
			
	Serum amyloid A	Increased	[176]
	Haptoglobin	Increased	[176]
	Circulating immune complexes	Conflicting results	[183,184]
Exhaled breath condensate			
	Methanol	Increased	[185]
	Ethanol	Increased	[185]

BALF—Bronchoalveolar lavage fluid; IFN-γ—gamma-interferon; MMP—matrix metalloproteinase.

Although APP can help in identifying local and systemic inflammation associated with sEA, they are not disease specific, and while SAA could potentially be associated with neutrophil recruitment, the role of these proteins in sEA needs to be better investigated.

The applicability of circulating immune complexes (CIC), which are formed by the union of an antigen and an antibody, has also been studied in sEA. These complex molecules were found to be increased in this disease [183,184] and, although conflicting reports question their diagnostic power, they may contribute to the monitoring of treatment response [184].

Similarly, matrix metalloproteinases (MMPs), tissue inhibitors of metalloproteinases (TIMPs) and MMPs/TIMPs ratio can be used to monitor disease severity and response to corticosteroids [181,182,186,187,188]. In general, MMPs are responsible for tissue destruction through collagen degradation [189], whilst TIMPs lead to the formation of fibrosis [190], and as such are thought to contribute to airway remodeling and fibrosis in chronic inflammation. The concentration of MMP-2, MMP-9, TIMP-1 and TIMP-2 decrease in response to treatment with corticosteroids [186], and with cytosine-phosphate-guanosine-oligodeoxynucleotides (CpG-ODN), an immunostimulatory drug [187]. However, since these biomarkers require invasive sampling, as they are measured in BALF, they are not suitable for evaluating treatment response.

IFN-γ has also been proposed as a biomarker of sEA, since it is increased in the BALF of these animals and is capable of distinguishing severely asthmatic horses from healthy individuals [172]. However, similarly to what is seen with MMP, it requires BALF sampling, rendering it unsuitable for repeated measurements.

In mEA-affected horses with airway neutrophilia (presenting more than 15% neutrophils in BALF), serum concentration of surfactant protein D (SPD) was reported to be significantly increased [191]. SPD could be a relevant biomarker for the diagnosis of EA, but additional research is required in severely asthmatic horses.

EBC is a non-invasive method which allows sampling of airway material and access to information about the metabolic status of the patient, even during disease exacerbation [165,185,192]. Preez et al. found that horses with lower airway inflammation have a higher pH and an increased hydrogen peroxide (H_2_O_2_) concentration in the air they exhale [192]. However, a different study reported no variations in the pH nor in the concentration of H_2_O_2_ between healthy horses and those with lower airway inflammation [165]. Both these studies did not focus exclusively on severely asthmatic horses and so it remains unclear whether these parameters could be of use as biomarkers of the disease. An additional study of metabolites in the EBC revealed that sEA-affected horses had increased concentrations of methanol and ethanol, compared to healthy controls [185]. The study of metabolomics in EBC has the potential to be a non-invasive approach to sEA diagnosis. Nonetheless, further research is necessary to better understand if this method has limitations, particularly if it can adequately distinguish between sEA-affected horses during remission and healthy individuals.

Current knowledge on sEA biomarkers is still limited and their use in a clinical context requires further research, since a noticeable benefit must be associated with these molecules in order for them to be included in the clinical guidelines for disease diagnosis and monitoring.

## 8. Microbiome 

The term microbiome refers to the community of microbes, such as bacteria, fungi, virus and archaea of a particular biological location [193,194]. These microorganisms interact functionally and metabolically, playing an important role in modulating the host’s innate immune response [195] and contributing to the inhibition of potential pathogens [196,197,198].

For many years it was mistakenly thought that the lung environment was sterile. However, the identification of microbiota in the lower respiratory tract of healthy humans has since discredited this belief [199,200,201,202].

Similarly, studies on equine microbiome have revealed that the same organizational taxonomic units (OTUs) can be found in the upper and lower airways of healthy individuals, although the latter anatomic region possessed an inferior biomass with decreased richness and diversity [203,204].

The phyla Proteobacteria, Firmicutes, Bacteroidetes, and Actinobacteria were found to be highly represented in healthy horses [203]. These phyla are also dominant in the lungs of healthy humans along with two other—Fusobacteria and Acidobacteria [199,200,205].

The microbiome is highly dependent on the host’s interaction with its environment. Thus, geographical location, housing conditions, diet, interactions with other individuals, and also treatment with antimicrobials and corticosteroids make each individual’s microbiome unique [204,206,207,208,209,210,211,212].

In humans, dysbiosis during infancy is considered a significant risk-factor for respiratory diseases such as asthma [213,214,215]. However, this relation has yet to be described in the equine population.

The lung microbiome of adult asthmatic people has been demonstrated to differ in both number and composition from that of healthy individuals, and these differences have been associated with airway hyperresponsiveness and obstruction [216,217]. Thus, the study of the lower airway microbiome in equine asthma stems from the hypothesis that these microbes play an important role in modulating the innate immune response of the host and may, therefore, contribute to the immunology of sEA [195].

The comparison of lung, nasal, and oral microbiomes of healthy and asthmatic horses showed that significant differences between groups could only be found in the lung microbiome at the taxonomic family level, with an overrepresentation of the Pasteurellacea family, but not at the phylum or OTU level, leading to the hypothesis that these differences were not inherent, but rather a consequence of inflammation [204].

Additionally, the bacteria *Nicoletella semolina*, a Pasteurellacea, has been detected in the upper and lower airways of both healthy and severely asthmatic horses [218,219,220]. Although its prevalence was increased in asthmatic horses, as detected by quantitative polymerase chain reaction (qPCR), no specific functional association between this bacteria and sEA was found [220], suggesting that it may be an opportunistic agent perpetuating airway inflammation in asthmatic animals.

Inversely, *Corynebacterium* spp. was commonly found in the trachea of a group of healthy horses, but its presence was decreased in the evaluated asthmatic group. As such, this microorganism could be a part of the normal microbiota of healthy horses, and might be one of the populations affected by the inflammatory changes in the airways [221].

Recent studies further point to the occurrence of dysbiosis in the lower airway microbiome of mEA-affected horses, although it is still unclear whether this results from persistent inflammation or chronic treatment with corticosteroids [203,221]. Furthermore, the relative abundance of *Streptococcus* was increased in mildly asthmatic horses, suggesting that the presence of this genus might be a risk-factor for mEA [203,222]. Similarly, infections with *Streptococcus pneumoniae*, and other opportunistic agents such as *Haemophilus influenzae* and *Moraxella catarrhalis*, are associated with acute asthma exacerbations in humans [223,224].

Fungi are often implicated in sEA exacerbation and are also considered as risk factors in human asthma [5,6]. Despite their relevance in disease pathophysiology, research focusing on the lung mycobiota of healthy and asthmatic horses is very limited. In healthy humans agents such as *Davidiellaceae* and *Cladosporium* [225], as well as *Eremothecium*, *Systenostrem*, and *Malassezia* [202], are the main contributors to the lung mycobiome. On the other hand, Charlson et al. have found that *Malassezia pachydermatis* is exclusively found in the BALF of asthmatic patients, which also showed significantly increased populations of *Termitomyces clypeatus* and *Psathyrella candollean* [225]. As for equine species, Bond and colleagues reported that the mycobiota of a group of mildly asthmatic horses comprised mainly of two phyla - Ascomycota and Basidiomycota [226]. Although a better characterization of healthy and asthmatic equine lung mycobiome is necessary, the reported results differ from those described in healthy humans [202,225]. Nonetheless, significant differences between the two species are to be expected, since stabling and hay feeding promote an environment rich in fungi [6].

To mitigate signs of airway inflammation and improve lung function, asthmatic patients are usually prescribed long-term treatment with corticosteroids. Because of their immunomodulatory effect, the use of corticosteroids can promote microbiome dysbiosis. In mEA-affected horses, the use of systemic dexamethasone affected the microbiota of the lower respiratory tract of healthy and asthmatic horses, increasing the relative abundance of 9 OTUs, including the abundance of *Streptococcus* spp. in the asthmatic group [203]. Similarly, the nebulization of dexamethasone resulted in an increase in the genera *Alysiella* and *Bordetella* in the lower respiratory tract, however this treatment had no effect on the population of *Streptococcus* found in the airways [226].

The relation between microbiome and corticosteroid is not unidirectional, since response to treatment is also influenced by lung microbiome. The microbiome of asthmatic humans diagnosed with corticosteroid resistance showed differences at the genus level compared to that of responsive patients. Furthermore, BALF AMs from asthmatic patients stimulated with *Haemophilus parainfluenzae*, a potential pathogen found in asthmatics with corticosteroid resistance, resulted in inhibition of response to corticosteroids, along with increased p38 mitogen-activated kinase phosphatase (MAPK) activation and increased *IL-8* and *mitogen-activated kinase phosphatase 1* (*MKP1*) mRNA expression. On the other hand, exposure to commensal *Prevotella melaninogenica* did not have a similar effect [227]. Reports also indicate that *H. parainfluenzae* can convert a Th2-type allergic asthma sensitive to corticosteroid treatment to a Th1 neutrophilic profile, with Il-17 expression [228]. Interestingly, Goleva and colleagues have also reported that inhibition of the transforming growth factor-β-associated kinase-1 (TAK1) in monocytes collected from the peripheral blood of asthmatic patients restored cellular sensitivity to corticosteroids [227], which could represent a novel therapeutic approach in patients which are refractory to these drugs. As such, the study of the microbiome can be useful in determining the response to therapy with corticosteroids.

Several studies have described how the gut microbiome of people with respiratory disease differs from that of healthy individuals [229,230,231], highlighting an immunological relationship between the lungs and gut. This interaction is termed the gut-lung axis, illustrating how these two distant anatomical sites appear to communicate. Not only does gut microbiota have a local immunological effect by interacting with the mucosal immune system [232], but it also produces pro and anti-inflammatory metabolites, such as biogenic amines (i.e., histamine), oxilipins, and short-chain fatty acids (SCFAs), which modulate the inflammatory response both locally and in the lung [233,234,235,236]. SCFAs reduce allergic response and airway inflammation in both humans and mice [237,238,239,240,241]. Trompette and colleagues reported that in an ovalbumin (OVA)-model, where mice were challenged with ovalbumin protein to induce an allergic inflammation in the lung, treatment with SCFAs increased the presence of dendritic cells with high phagocytic ability but with impaired capacity of activating Th2 effector cells in the lungs [240].

Allergic diseases are also associated with a lower fecal microbial diversity in humans, and, although these findings have mostly been reported in infants, they have also been described in adults [242].

Differences in the fecal microbiome of asthmatic (mEA) and healthy horses appeared to occur mostly during disease exacerbation [207]. The reported dysbiosis was observed using an OTU analysis approach and was found to be accompanied by an increased representation of the Firmicutes phylum, namely Clostridia class [207]. These authors hypothesized that lung inflammation and compromised oxygenation would induce changes in the gut microenvironment, since fewer differences were observed when both groups of horses were at pasture, a less pro-inflammatory environment. However, no causative relation was established and disease remission could also be secondary to the changes induced in the gut microbiome by leaving the horses out to pasture. Dysbiosis of bacteria belonging to the phylum Firmicutes has also been documented in several studies on gut and respiratory microbiome of asthmatic humans [243,244] and further research could prove to be of interest in equine asthma.

Conversely, Kaiser-Thom and colleagues compared the fecal microbiota of horses diagnosed with either sEA, culicoides hypersensitivity or both to that of healthy individuals using a Divisive Amplicon Denoising Algorithm (DADA2) approach to analyze microbial taxonomy, and found no significant differences between the microbe populations [206].

Research is currently focused on alternative therapies which could revert the dysbiosis observed in asthmatic individuals and thus impacting immune responses. Since treatment with antibiotics is not a viable option, supplementation with probiotics and soluble fiber are currently under investigation [238,245]. In an OVA-induced mouse model of allergic airway inflammation, the use of probiotics induced regulatory T cells differentiation and suppressed Th2 allergic response [245]. Further studies are necessary to understand if horse gut microbiome could also benefit from such treatments and whether they would in fact result in the modulation of the inflammatory response associated with sEA.

The microbiome of asthmatic humans has been associated with specific disease endotypes. For example, increased representation of Proteobacteria is found in severe asthma with neutrophilic exacerbation [246] and Th17-related gene expression [247]. As such, study of the microbiome can further enable the practice of precision medicine, and increase the likelihood of a good prognosis in asthmatic patients since it will allow a personalized therapeutic approach. However, these interactions have yet to be described in equine medicine.

Studies on the microbiome of asthmatic horses are mostly descriptive, portraying the microbial populations found in the respiratory and intestinal tract. Although some studies do characterize the degree of airway inflammation of these horses by using BALF cytology and lung function evaluation, cross-referenced data with existing cytokine profile is, nonetheless, currently missing. Whether equine pulmonary microbiome will differ according to asthma endotype and inflammatory cell population remains to be ascertained.

Another limitation is the small number of animals enrolled in each study which limits the statistical significance of the results, influences the conclusions and may further impair the establishment of causality. Additionally, in most studies disease exacerbation was achieved by altering the horses’ environment and diet which inevitably influences the microbiome of the studied horses and works as a confounding factor in the interpretation of results. Furthermore, it is still not clear to which extent the altered microbiome causes, perpetuates or is secondary to airway inflammation in equine and human asthmatic patients.

Further research on the microbiome of equine asthmatics will undoubtably contribute to the elucidation of the current loopholes in this subject.

## 9. Conclusions and Future Directions

Further characterization of the disease’s genetic background is fundamental to improve current knowledge of the pathways involved in the heritability and expression of sEA. The reported genetic research focuses mostly on a well characterized subpopulation of Swiss Warmblood horses which limits the applicability of these findings to the general horse population. Nonetheless, the reported genetic heterogeneity and complexity observed in the above mentioned families likely occurs in other individuals, although, potentially, other genes and pathways may be involved. Thus, research on a large multi-center population of client owned sEA-affected horses with a detailed genetic background is needed to further contribute to the description of the genetic events that take place in these animals.

Although the clinical phenotype of sEA has been thoroughly described [1], the immunological mechanisms which lead to inflammation and structural changes in the airways (mucus accumulation, bronchial constriction and bronchial wall thickening) still lack clarification. Several cytokines, chemokines and inflammatory cells participate in the pathogenesis of asthma; however their precise characterization is still unclear. The inconsistencies found between reported studies may arise from differences in their experimental design and methodologies. Furthermore, the limited number of animals included in these works may hinder the attainment of significant results.

Thus, cooperation between research groups and research based on large multi-center populations of client owned sEA-affected horses could potentially solve some of these limitations. Also, uniformization of methodologies and protocols will enable the comparison of reported results, allowing a better definition of the genetic and immune mechanisms associated with sEA.

Additionally, more encompassing studies using genomic, transcriptomic, proteomic and metabolomic analysis will undoubtably enhance the scientific knowledge of the disease. This will enable an understanding of how genetics can determine cytokine and chemokine expression and how these proteins and metabolites influence disease expression. Furthermore, the impact of lung and gut microbiome also needs to be assessed, since these microbes regulate the immune innate response from an early age and can also promote airway hyperreactivity and inflammation in asthmatic individuals.

Although sEA shares many similarities with its human counterpart, an understanding of this disease based on the extrapolation of reported data for other species is unfeasible. Thus understanding the origin of equine AMs and the characterization of this population is necessary to recognize how these cells influence the inflammatory pathways of asthmatic horses. 

Immunological and genetic characterization will likely assist in the identification of disease endotypes and more importantly contribute to the development of novel therapeutic targets. For example, anti-interleukin targeted therapies, using monoclonal antibodies, could help manage the disease, especially in horses with resistance to corticosteroids. This is currently being researched in human asthma, where identification of disease endotypes associated with specific cytokine profiles has led to the development of monoclonal antibodies. One such example is Tralokinumab, a human anti-IL-13 monoclonal antibody for uncontrolled asthma [248].

Since the precise cytokine profiles of equine asthma are not fully understood, current research is focusing on identifying the causal allergens which trigger airway inflammation [5], and how immunotherapy can help modulate the inflammatory response with promising results [9,187]. Also, determining specific allergen susceptibility can contribute to the development of specific immunotherapy and, in theory, help devise environmental management protocols for affected horses.

Novel diagnostic tools based on genetics or disease biomarkers would prove of significant value to equine medical practitioners, especially if they are able to positively identify a severely asthmatic horse during remission. Current diagnosis relies mostly on invasive methods which are not suitable for evaluating treatment response, since it will require repeated measurements. Thus, systemic blood biomarkers and exhaled breath condensate are attractive alternatives to BALF sampling. Research should focus on defining cutoff levels and constructing a panel of biomarkers which could substitute BALF cytology when monitoring treatment response.

In conclusion, current research shows that the genetic and inflammatory pathways involved in sEA are complex and variations are to be expected between subsets of individuals. A deeper knowledge of the disease’s immunological pathways will allow the definition of endotypes, the detection of inflammatory biomarkers of diagnostic value, and a personalized therapeutic approach targeting the inflammatory pathways involved in the disease.

## Figures and Tables

**Table 1:** Cytokines reported in sEA-affected horses according to T helper subtype [10,43,49,52,56,57,58,59,60,61,62].

Th2	Th17	Th1/Th2	Th1/Th17	Th2/Th17	Undefined
↑ IL-4 ^1(r)^	↑ CXCL13 ^2(r)^	↑ IL-4 ^1(r)^	↑ IL-1β^1(r)^	↓ miR-197 ^2(r)^	↓ IFN-γ ^1(r)^
↑ IL-5 ^1(r)^		↑ IFN-γ ^1(r)^	↑ IL-8 ^1(r); 3(r); 3(p)^	↑ miR-744 ^2(r)^	↓ IL-4 ^1(r)^
↓ IFN-γ ^1(r)^			↑ IFN-γ ^1(r)^	↓ miR-26a ^4(r)^	↓ IL-5 ^1(r)^
			↑ TNF-α ^1(r)^	↑ miR-31 ^4(r)^	↓ IL-13 ^1(r)^
			↑ IL-17 ^1(r)^	↓ TNF-α ^4(r)^	
				↑ IL-4R ^4(r)^	

^1^—BALF recovered cells; ^2^—Peripheral blood; ^3^—bronchial epithelial biopsy; ^4^—Lung tissue (post-mortem). r—RNA detected; p—protein detected. ↑—increased expression; ↓—decreased expression. IL—interleukin; IL-4R—interleukin 4 receptor; IFN-γ—gamma-interferon; TNF-α—tumor necrosis factor-α; miR—microRNA.

## Data Availability

Not applicable.
